# Preliminary study on mesenchymal stem cells in repairing nerve injury in pelvic floor denervation

**DOI:** 10.3389/fbioe.2023.1190068

**Published:** 2023-06-22

**Authors:** Guorui Zhang, Yuxin Dai, Jinghe Lang

**Affiliations:** Department of Obstetrics and Gynecology, State Key Laboratory of Complex Severe and Rare Diseases, National Clinical Research Center for Obstetric and Gynecologic Diseases, Peking Union Medical College Hospital, Chinese Academy of Medical Sciences and Peking Union Medical College, Beijing, China

**Keywords:** mesenchymal stem cell, pelvic organ prolapse, pelvic floor dysfunction, nerve injury, stem cell transplantation

## Abstract

**Introduction:** Nerve injury is considered one of the causes of pelvic floor dysfunction. Mesenchymal stem cells (MSCs) transplantation provides new possibilities for refractory degenerative diseases. This study aimed to explore the possibility and strategy of mesenchymal stem cells in treating pelvic floor dysfunction nerve injury.

**Methods:** MSCs were isolated from human adipose tissue and cultured. A MSCs suspension (40 µL at 5 × 107/mL) was loaded on a gelatin scaffold. A rat model of anterior vaginal wall nerve injury was established by bilateral pudendal nerve denervation. The nerve tissue repair effect of mesenchymal stem cells transplanted into the anterior vaginal wall of a rat model was explored and compared in the following three groups: blank gelatin scaffold group (GS group), mesenchymal stem cell injection group (MSC group), and mesenchymal stem cells loaded on the gelatin scaffold group (MSC-GS group). Nerve fiber counting under a microscope and mRNA expression of neural markers were tested. Moreover, mesenchymal stem cells were induced into neural stem cells *in vitro*, and their therapeutic effect was explored.

**Results:** Rat models of anterior vaginal wall nerve injury induced by bilateral pudendal nerve denervation showed a decreased number of nerve fibers in the anterior vaginal wall. qRT-PCR revealed that the content of neurons and nerve fibers in the rat model began to decrease 1 week after the operation and this could continue for 3 months. *In vivo* experiments showed that MSC transplantation improved the nerve content, and MSCs loaded on the gelatin scaffold had an even better effect. mRNA expression analysis demonstrated that MSCs loaded on gelatin scaffolds induced a higher and earlier gene expression of neuron-related markers. Induced neural stem cell transplantation was superior in improving the nerve content and upregulating the mRNA expression of neuron-related markers in the early stage.

**Conclusion:** MSCs transplantation showed a promising repair capacity for nerve damage in the pelvic floor. The supporting role of gelatin scaffolds might promote and strengthen the nerve repair ability at an early stage. Preinduction schemes could provide an improved regenerative medicine strategy for innervation recovery and functional restoration in pelvic floor disorders in the future.

## 1 Introduction

Pelvic floor dysfunction (PFD) is a cluster of diseases caused by defects in pelvic floor support structures, including pelvic organ prolapse (POP), stress urinary incontinence (SUI), chronic pelvic pain and sexual dysfunction, which are common problems among middle-aged and elderly women. The main risk factors for PFD include childbirth injury, obesity and old age (Vergeldt et al., 2015). Other possible reasons include smoking, increased intra-abdominal pressure (chronic cough or constipation), estrogen deficiency, connective tissue disease and genetic tendency. A decrease in pelvic floor function caused by nerve injury is considered one of the pathogeneses of PFD (Kuo, 2000).

Previous studies have confirmed that damage to the pelvic floor nerves, abnormal peptide neurotransmitters, and denervation of pelvic floor muscles are common in PFD, revealing that pelvic floor nerve injury is an important mechanism in the occurrence and development of PFD. Zhu (Zhu et al., 2004) found that the content of nerve fibers (by immunohistochemical staining of protein gene product 9.5 (PGP 9.5)) in the anterior vaginal wall of the POP group and SUI group was significantly lower than the control group. With the aggravation of POP symptoms, the contents of neuropeptide Y (Hu et al., 2012) and vasoactive intestinal peptide (Hu et al., 2013) in anterior vaginal wall tissue gradually decreased. With a better understanding of the high-risk factors for PFD, the nerve injury mechanism of pelvic floor supporting structures has become a new hot spot for etiological research on PFD and will pave the way for the development of more effective treatment options.

The development of tissue engineering and regenerative medicine provides new possibilities for refractory degenerative diseases (Ma et al., 2021). Considering the strong correlation between PFD and connective tissue injury, mesenchymal stem cells (MSCs) have great potential as seed cells (Lin et al., 2022). Previous studies have confirmed its safety and possible effectiveness (Mukherjee et al., 2019). Zou XH (Zou et al., 2010) found that MSCs loaded with sling transplantation could repair collagen tissue in the anterior vaginal wall of an SUI model in rats and increase the pressure of the bladder leakage point. In Ulrich’s study (Ulrich et al., 2014), human endometrial stem cells were cocultured with gelatin-coated polyamide mesh to make tissue engineering mesh, which was implanted under the skin of the back of nude mice. Mao’s study believed MSCs normalized the fibromuscular structures of the vaginal wall of ovariectomized rats potentially through a paracrine effect (Mao et al., 2021). Exosomes, one of the major secretions of MSCs, could modify fibroblast activation and secretion, facilitate extracellular matrix modelling, and promote cell proliferation to enhance pelvic tissue regeneration in PFD (Xu et al., 2023).

Above all, MSCs show potential in promoting connective tissue regeneration and anti-inflammatory reactions. However, various complicated factors are involved in the complex chronic tissue injury represented by PFD, and the existing technology is still far from ready for clinical applications. Moreover, recent studies have demonstrated that MSC-based therapy significantly improves nerve repair in neural injury or degenerative diseases, including retinal degenerative diseases (Holan et al., 2021), traumatic brain injury (Wang et al., 2022) and peripheral nerve injury (Zhang et al., 2021). Whether MSCs could promote nerve repair in pelvic floor nerve injury remains unexplored.

**FIGURE 1:**
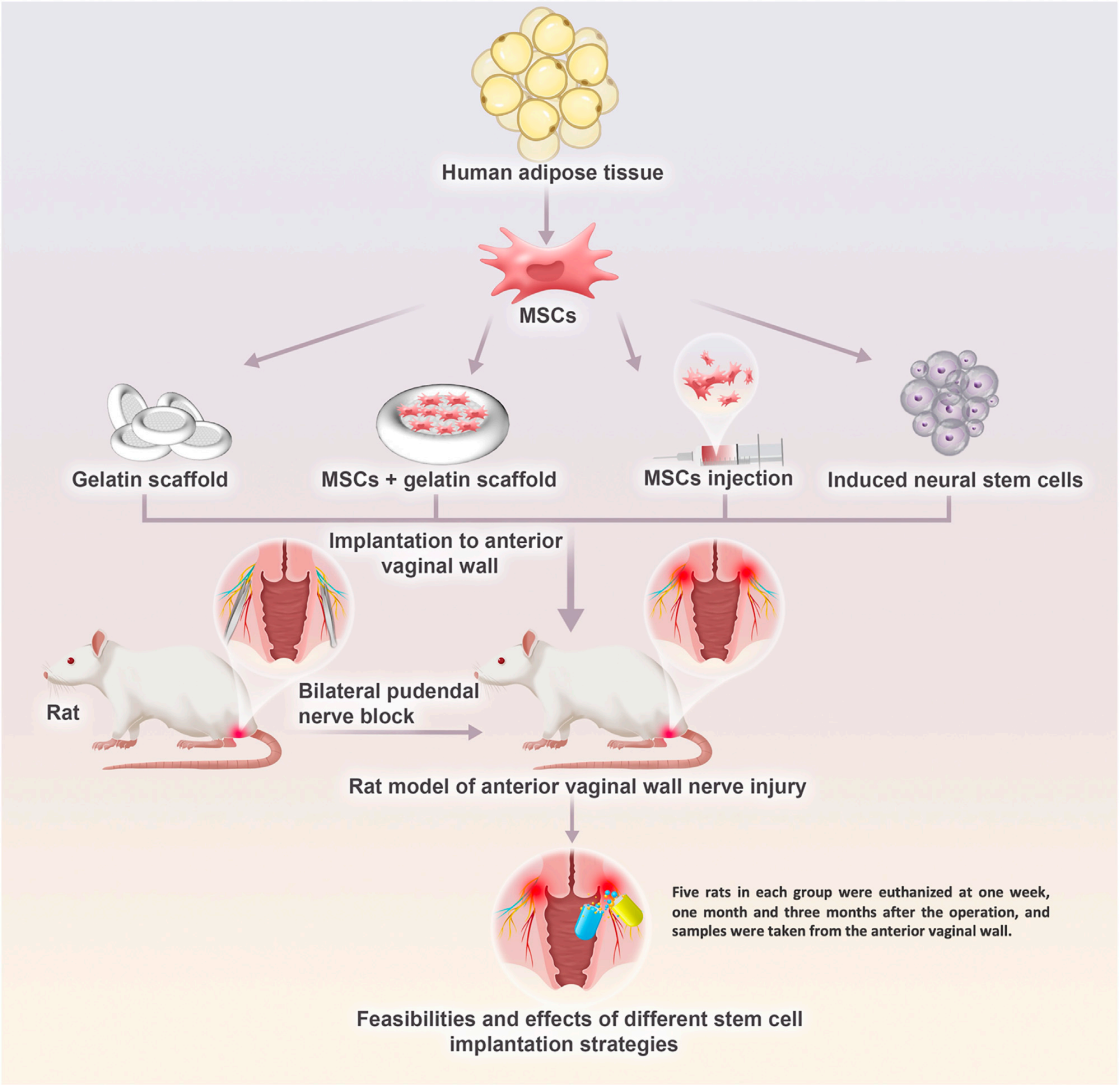
The research plan for the development of a potential therapeutic solution of regenerative medicine strategy for innervation recovery and function restoration in pelvic floor disorders in the future. Pelvic nerve damage rat model by bilateral pudendal nerve denervation was treat with blank gelatin scaffold transplantation, mesenchymal stem cells injection, and mesenchymal stem cells transplantation loaded on the gelatin scaffold. What’s more, therapeutic effect of induced neural stem cells from mesenchymal stem cells were also evaluated.

To further explore the possibility of treating PFD by repairing injured nerves, we constructed a model of pelvic nerve injury in rats by bilateral pudendal nerve denervation. The feasibility and the effects of different stem cell implantation strategies were studied, which provided a preliminary basis for future comprehensive research (Figure 1).

## 2 Methods

### 2.1 Isolation, passage and identification of MSCs

Adipose tissue was obtained after liposuction in the Plastic Surgery Department of Peking Union Medical College Hospital, Chinese Academy of Medical Sciences, from young women aged 20–40. Tissue samples were added to 0.2% type I collagenase at a ratio of fat: collagenase = 3:1, digested for 30 min in a shaker at 37°C at 1,000 r/min, and filtered with a 100 mesh sieve to remove undigested tissue. After centrifugation at 1,500 rpm for 10 min, the cells were resuspended in MSC medium at a density of 1 × 106/mL and cultured in a cell incubator at 37°C and 5% carbon dioxide. The culture medium was changed every 2 days until the cell growth density was approximately 80%, and then the cells were passaged.

The immunophenotype of the MSCs was detected by flow cytometry after indirect immunofluorescence staining. Well-growing third-generation cells were collected, digested with 0.125% trypsin, incubated with primary antibodies against CD105, CD29, CD34, CD31, CD44, CD106, HLA-DR, and Flk1 at 4°C for 30 min, and then incubated with a FITC-labeled secondary antibody in the dark at 4°C for 30 min.

### 2.2 Induction of MSCs into neural stem cells *in vitro*

Human adipose-derived mesenchymal stem cells were induced and differentiated into neural stem cells (NSCs) by adding 9 small molecules (Supplementary Material S1.2) as previously described in the literature (Zhang et al., 2016). Neural stem cell-inducing medium was prepared by adding the 9 small molecules (see the Supplementary Material for the name, working concentration, molecular weight and function). The 3rd-5th generation human adipose-derived mesenchymal stem cells were inoculated into a 12-well plate at a density of 20,000 cells/well and cultured overnight in a cell incubator at 37.0°C, 5% carbon dioxide and 20% oxygen until the mesenchymal stem cells adhered to the well, and then the growth medium was replaced with induction culture medium, and the cells were observed every day. After induction culture for 12 days, the cells were inoculated into suspension dishes to observe the morphology of the neural stem cells in suspension culture. The expression of the Sox2, Pax6, Nestin, Olig2, and Ascl1 genes in cells induced at D0, D2, D4, D6, D8, D10, D12, D14, and D16 was detected by qRT-PCR (see the Supplementary Material for the primer sequences), and the expression of the neural stem cell marker proteins Sox2, Pax6, Nestin, Olig2, and Ascl1 was detected at the protein level by Western blot.

### 2.3 Preparation of cells loaded on gelatin scaffolds

When the MSCs subcultured to the 3rd-4th generation reached 80% confluence, they were removed from the dish with 0.125% trypsin digestive solution and loaded on the gelatin scaffolds (GoMatrix, a gelatin 3-dimensional scaffold with pore size 90 um) in a 48-well plate under aseptic conditions. The cells were adjusted to 5×10^7^/mL and 40 µL was dropped onto each gelatin scaffold. The MSCs and gelatin scaffolds were incubated in a 37.0°C, 5% carbon dioxide cell incubator for 4 h so that the cell suspension could fully penetrate into the gelatin scaffold. Then, 500 µL of MSC culture medium was added to each well of the 48-well plate and it was placed in a 37.0°C, 5% carbon dioxide cell incubator overnight. These scaffolds with evenly distributed MSCs inside were then applied to transplantation *in vivo*.

### 2.4 Rat model of anterior vaginal wall nerve injury induced by a bilateral pudendal nerve denervation

Forty-five 10-week-old female SD rats weighing 220 ± 15 g were randomly divided into three groups: normal group (5 rats), sham operation group (SO group) (20 rats) and bilateral pudendal nerve denervation group (BPND group) (20 rats). The normal group was not treated; the BPND group underwent bilateral pudendal nerve denervation; the SO group underwent similar operations, but the bilateral pudendal nerves were only dissociated and not removed. The pudendal nerve originated from the sacral plexus and carried sensory, motor, and autonomic nerve fibers, mainly distributed in the pelvic floor tissue. The procedures of the pudendal nerve denervation were as follows: the skin was incised from the midline of the back near the buttock side, separating the subcutaneous fascia and exposing the gap between the lateral femoral muscle and adductor magnus muscle. Cutting upward along the lumbosacral trunk, two nerve branches could be seen near the spine, and the lateral branch is the pudendal nerve (Supplementary Figure S1), accompanied by the internal pudendal artery and vein. The pudendal nerve was dissociated 8–10 mm from the beginning and 2–3 mm was then cut off.

Rats in the normal group were euthanized at the beginning of the experiment and their anterior vaginal walls were sampled. The rats in the sham operation group and animal model group were euthanized at 3 days, 1 week, 1 month or 3 months after the operation. Five rats in each group were sampled at each time point. The anterior vaginal wall tissues were paraffin-embedded, sliced, stained with hematoxylin and eosin, and stained with anti-PGP9.5 antibody as the primary marker of nerve fibers. The number of nerve fibers in each visual field was counted under a microscope, and a total of 10 high-power visual fields were counted in each section.

The vaginal anterior wall tissue was crushed, total RNA was extracted (refer to the TRIzol instructions of Invitrogen Company), cDNA synthesis was carried out according to M-MLV product instructions, and real-time PCR was conducted. After predenaturation at 94°C for 10 min, 40 cycles were started: denaturation at 94°C for 15 s and annealing at 60°C for 40 s. With glyceraldehyde-3-phosphate dehydrogenase (GAPDH) as an internal reference, the relative value of mRNA expression in each group was calculated by the 2^−ΔΔCt^ method, and the mRNA expression levels of UCHL, Rbfox3, Tubb3, MAP2, NSE, Noggin, Nestin, Sox2, and Vimentin were detected (see the supplementary material for the primer sequences).

### 2.5 Exploration of the nerve tissue repair effect of MSCs transplanted into the anterior vaginal wall of a rat model

Forty-five 10-week-old female SD rats weighing 220 ± 15 g were randomly divided into three groups: a) blank gelatin scaffold group (GS group), in which a gelatin scaffold without cells was implanted into the anterior vaginal wall (a 5 mm long incision at the outer opening of the anterior vaginal wall in rats was made with a blade, and the submucosal layer was dissociated bluntly to the top end of vagina, and then a gel scaffold was placed into the submucosal layer in the middle part of anterior vaginal wall, and finally the incision was sutured); b) mesenchymal stem cell injection group (MSC group), in which a mesenchymal stem cells suspension was injected into the anterior vaginal wall (fine needle of the syringe was inserted from the external opening into the submucosal layer of the anterior vaginal wall, with a depth of 1.5 cm, and cell suspension was injected accompanied by needle withdraw till the external opening of the vagina wall, with a total cell suspension volume of 500 ul, at a cell concentration of 4×10^6^/mL); and c) mesenchymal stem cells loaded on the gelatin scaffold group (MSC-GS group), in which mesenchymal stem cells loaded on a gelatin scaffold were transplanted into the anterior vaginal wall (the same procedure as the GS group, with gelatin scaffold loaded with stem cells implanted). Bilateral pudendal nerve denervation was performed on rats in each group. After bilateral PNB, gelatin scaffolds, MSC suspensions (1 × 107/mL, 200 µL) or MSCs loaded on gelatin scaffolds were transplanted into the anterior vaginal wall according to the above methods. Five rats in each group were euthanized at 1 week, 1 month and 3 months after the operation, and samples were taken from the anterior vaginal wall. Nerve fiber counting under a microscope was performed after PGP9.5 immunohistochemical staining. The mRNA expression levels of UCHL, Rbfox3, Tubb3, MAP2, NSE, Noggin, Nestin, Sox2 and Vimentin were detected by qRT-PCR.

### 2.6 Statistical analysis

All data are expressed as the mean standard deviation ( $\bar{x}$ ± S), and comparative analysis of nerve fibers and relative gene expression were performed by ANOVA. *p* < 0.05 was defined as a significant difference. All procedures and protocols were approved by the Interstitial Committee of Peking Union Medical College Hospital.

## 3 Results

### 3.1 Isolation and identification of MSCs

**FIGURE 2:**
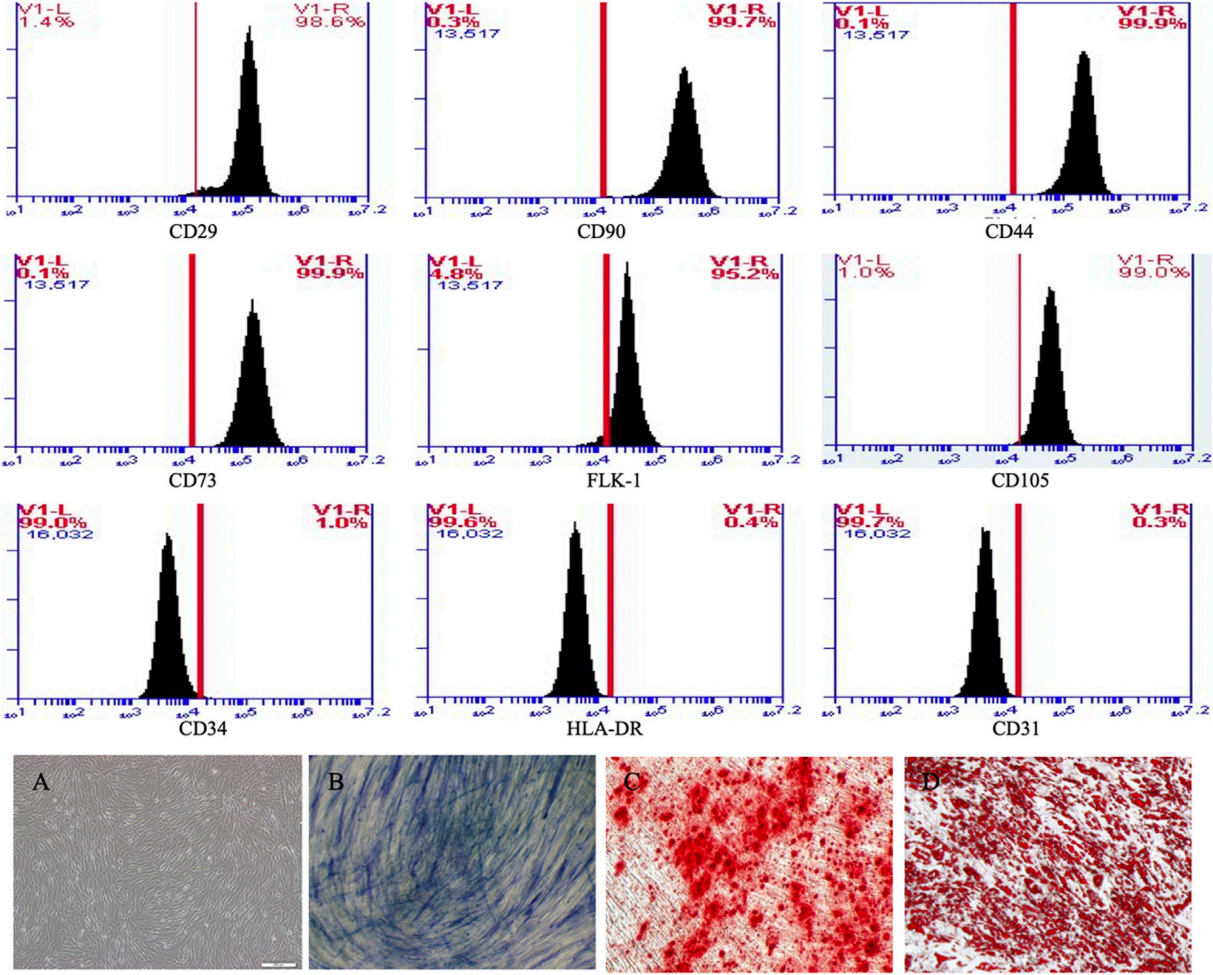
MSCs expressed CD29, CD90, CD44, CD73, FLK-1 and CD105, but not CD34, HLA-DR and CD31 in flow cytometry. **(A)** MSCs were spindle-shaped and vortex-lined. **(B)** After 8 days osteogenic induction, most cells were stained blue with alkaline phosphatase and **(C) **red with alizarin red. **(D)** After 8 days adipogenic induction, most cells were stained red with oil red.

From the 3rd to the 5th generation, cultured MSCs were spindle-shaped and vortex-lined (Figure 2A). The expression of the membrane markers of the MSCs detected by flow cytometry showed that the cells expressed CD29, CD90, CD44, CD73, FLK-1, and CD105 but not CD34, HLA-DR or CD31 (Figure 2). To verify their differentiation capacity, the third generation of MSCs was induced by osteogenic and lipogenic culture medium. The cells gradually began to secrete mineralized matrix after culture in osteogenic medium. After 8 days of induction, white mineralized nodules were observed under a microscope, and most cells were stained blue with alkaline phosphatase (Figure 2B) and red with Alizarin red (Figure 2C). These results showed that the extracted human adipose-derived MSCs could differentiate into osteoblasts. Round and transparent lipid droplets gradually appeared in the cells cultured in lipogenic medium, which gradually increased and merged into large lipid droplets. After induction culture for 9 days, the cells were stained red with Oil red O, demonstrating the ability to differentiate into adipocytes (Figure 2D). These results indicated that the cells we isolated were MSCs with multiple differentiation potential. We selected 3rd-to 5th-generation cells for subsequent experiments.

### 3.2 Induction of MSCs into neural stem cells

In addition to their secretion mechanism, the strong differentiation ability of MSCs has also been widely studied. MSCs are not normally located upstream of nerve cells in the differentiation process. However, a large number of studies have confirmed that MSCs can promote the process of nerve repair through differentiation (Holan et al., 2021). In our *in vivo* experiments, the early high expression of neural stem cell markers in the MSC group might be related to this mechanism. To study the correlation between this mechanism and treatment, in this part of the work, we actively induced MSCs into neural stem cells to observe their curative effect.

**FIGURE 3:**
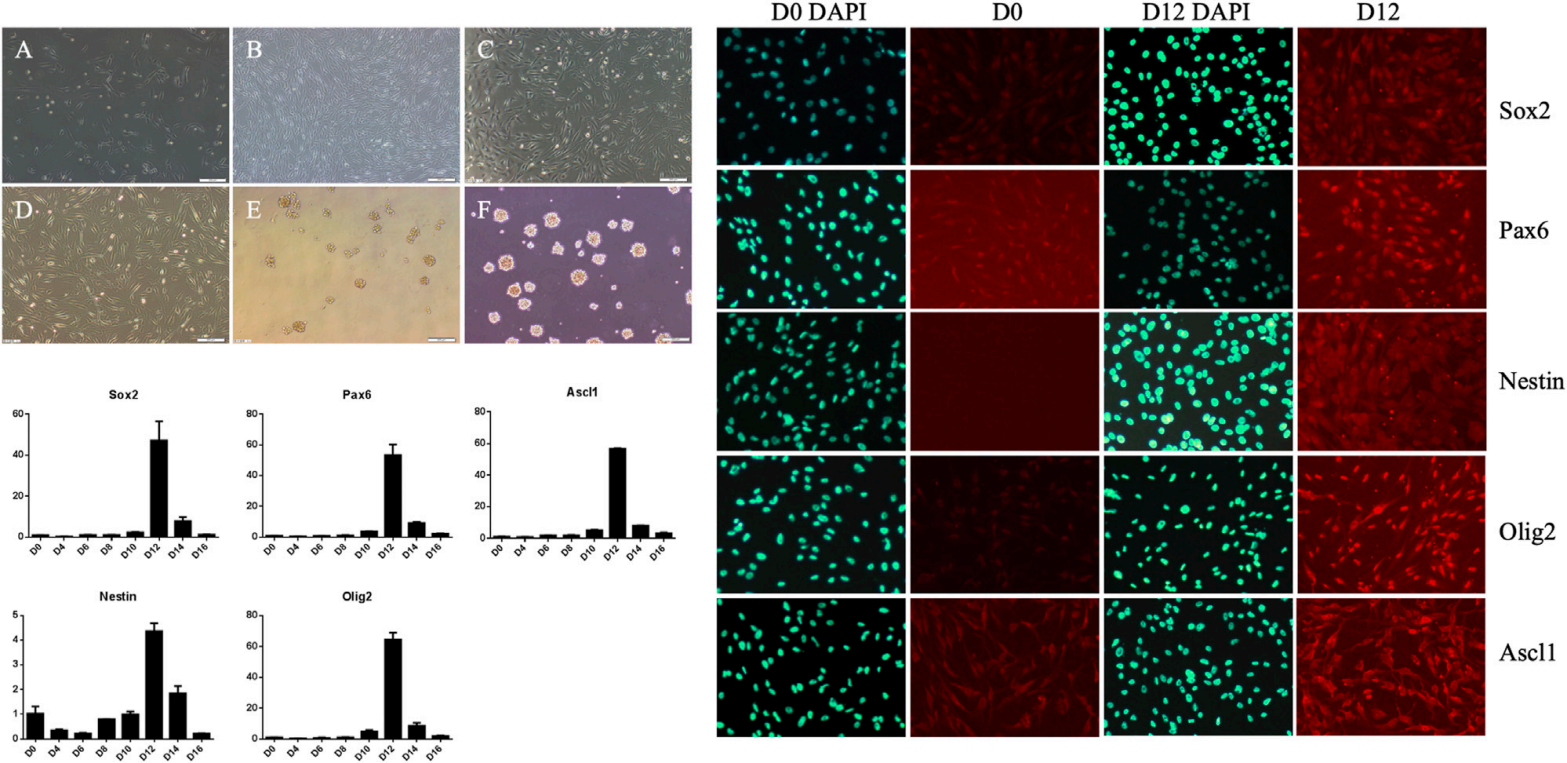
Morphological changes of mesenchymal stem cells during neural stem cell induction culture and expansion culture, pre-induction cells **(A)**, induction for 6 days **(B)**, induction for 12 days **(C)**, induced cells re-adhered to the wall and expanded for 1 day **(D)**, suspension culture for 1 day **(E)**, suspension culture for 4 days **(F)**. The expressions of Sox2, Pax6, Ascl1, Nestin and Olig2 in D0, D2, D4, D6, D8, D10, D12, D14 and D16 cells were detected by qRT-PCR, reaching the peak at D12. The expressions of Sox2, Pax6, Nestin,Olig2 and Ascl1 in cells before induction (D0) and on day 12 (D12) were detected by cell fluorescence.

Human adipose-derived MSCs underwent certain morphological changes under the action of neural induction medium (Figure 3). In the first 6 days, promotion of cell proliferation was the main change. The number of cells increased, but morphological changes were not obvious and they remained spindle-shaped and arranged in a vortex. After 6 days, cell proliferation slowed down, and their cell morphology gradually changed. By the 12th day of induction culture, both ends of the cells gradually became blunt and round, the volume of cells increased slightly, and the refractive index of the cell edge increased. After induction for 12 days, the cells were digested by trypsin and cultured in neural stem cell amplification medium. When the cells were inoculated in gelatin-coated dishes, the cells adhered to the dish and grew. When the cells were inoculated in low adhesion dishes, the cells grew in suspension, proliferated and aggregated to form suspended cell spheres, that is, neural stem cells. The growth in suspension as spheroids is one of the growth and morphological characteristics of neural stem cells. To further verify the induction of neural stem cells, we collected uninduced human adipose-derived MSCs and neural stem cells induced for 12 days. After immunofluorescence labeling, we found that the expression of Sox2, Pax6, Nestin, Olig2, and Ascl1 increased significantly in induced cells (Figure 3). At the same time, we also detected gene expression. The results showed that the expression of Sox2, Pax6, Nestin, Olig2, and Ascl1 increased gradually after induction and reached a peak at Day 12 (Figure 3). These results showed that MSCs could become neural stem cell-like cells after induction, which supported our conjecture that MSCs could directly repair neural injury and put forward a new idea for the following treatment plan.

### 3.3 Establishment and identification of anterior vaginal wall nerve injury rat models

**FIGURE 4:**
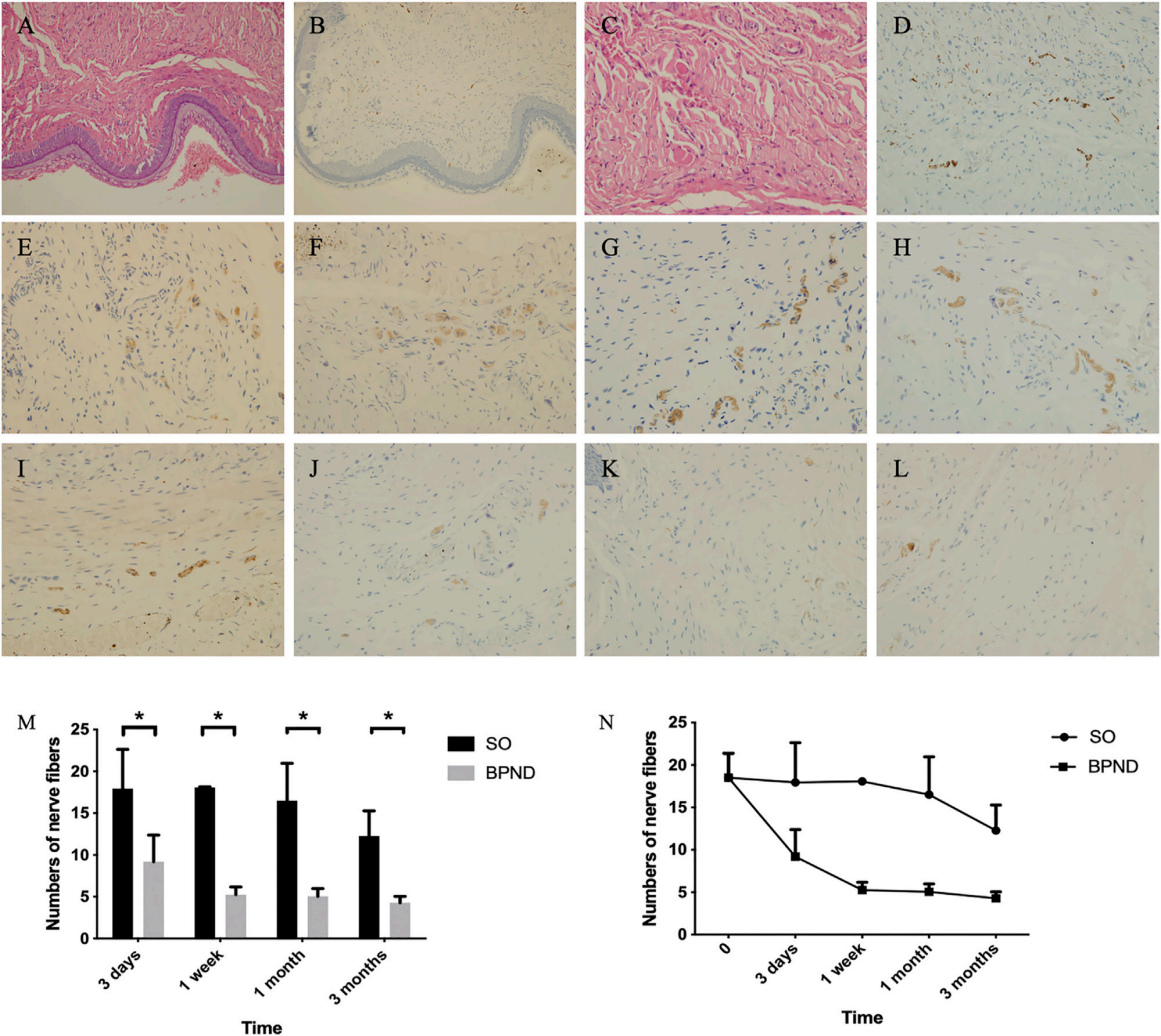
Distribution of nerve fibers in anterior vaginal wall of rats in each group shown by hematoxylin-eosin (HE) staining and UCHL immunohistochemical staining. HE staining of the anterior vaginal wall of normal rats was shown in **(A)** (200 ×) and **(C)** (400 ×), and nerve fibers distribution in **(B)** (200 ×) and **(D)** (400 ×). Density of nerve fibers were shown at 3 days **(E)**, 1 week **(F)**, 1 month **(G)** and 3 months **(H)** after operation in the sham operation group; at 3 days **(I)**, 1 week **(J)**, 1 month **(K)** and 3 months **(L)** after operation in bilateral pudendal nerve denervation group. The comparison **(M)** and changes **(N)** and of nerve density in anterior vaginal wall at different time after operation in normal group, sham operation group (SO) and bilateral pudendal nerve denervation group (BPND). * the difference was statistically significant (*p* < 0.05).

There are many small peripheral nerves scattered in the anterior vaginal wall of rats. Ubiquitin C-terminal hydrolase L (UCHL), a specific nerve fiber index, is widely distributed in nerve cell bodies and axons. Nerve fiber counting with UCHL staining in the anterior vaginal wall of normal rats was 18.5 ± 2.9 per high-power field (HPF). Compared with the normal and SO groups, the BPND group showed a significantly decreased number of nerve fibers in the anterior wall of the vagina after the operation, and this effect could last for 3 months (Figure 4; Supplementary Table S1). This suggested that the number of nerve fibers in the anterior vaginal wall of rats decreased after bilateral pudendal nerve denervation.

**FIGURE 5:**
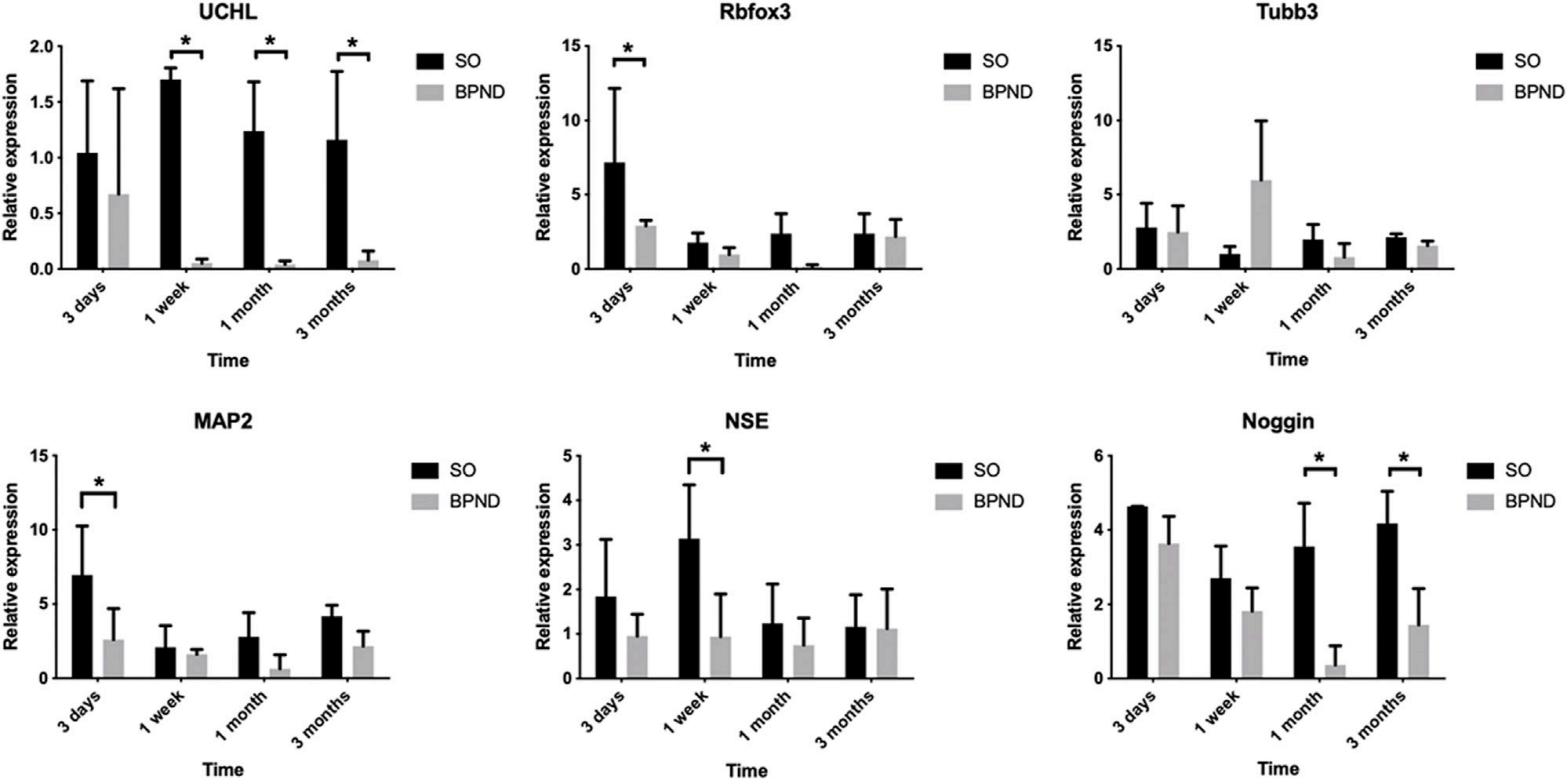
The expressions of UCHL, Rbfox3, Tubb3, MAP2, NSE, noggin, nestin and Sox2 in the anterior vaginal wall of sham operation group (SO) and bilateral pudendal nerve denervation group (BPND) were detected by qRT-PCR. * the difference was statistically significant (*p* < 0.05).

Neural-related mRNA expression analysis by qRT-PCR (Figure 5) showed that UCHL did not change significantly in the SO group but it decreased significantly at 1 week and 1 month after the operation in the BPND group and recovered to a certain extent 3 months after the operation. The mature neuron marker Rbfox3 showed no significant difference between the two groups at 1 week and 3 months but decreased significantly 1 month after the operation. There was no significant difference between the neuron axon marker Tubb3 and the dendrite marker MAP2 in the SO and BPND groups. The expression of the early neuron marker Noggin in the BPND group was similar to that in the SO group at the early stage (3 days–1 week) and significantly lower than that in the SO group at the later stage (1 month–3 months). These results suggested that after bilateral pudendal nerve denervation, the content of neurons and nerve fibers in the anterior vaginal wall of rats began to decrease 1 week after the operation and this effect could last for 3 months. We selected four nerve-related genes with different degrees of significant changes, including UCHL, Rbfox3, NSE, and Noggin, as markers for evaluation of the transplantation treatment.

### 3.4 Evaluation of MSC transplantation

**FIGURE 6:**
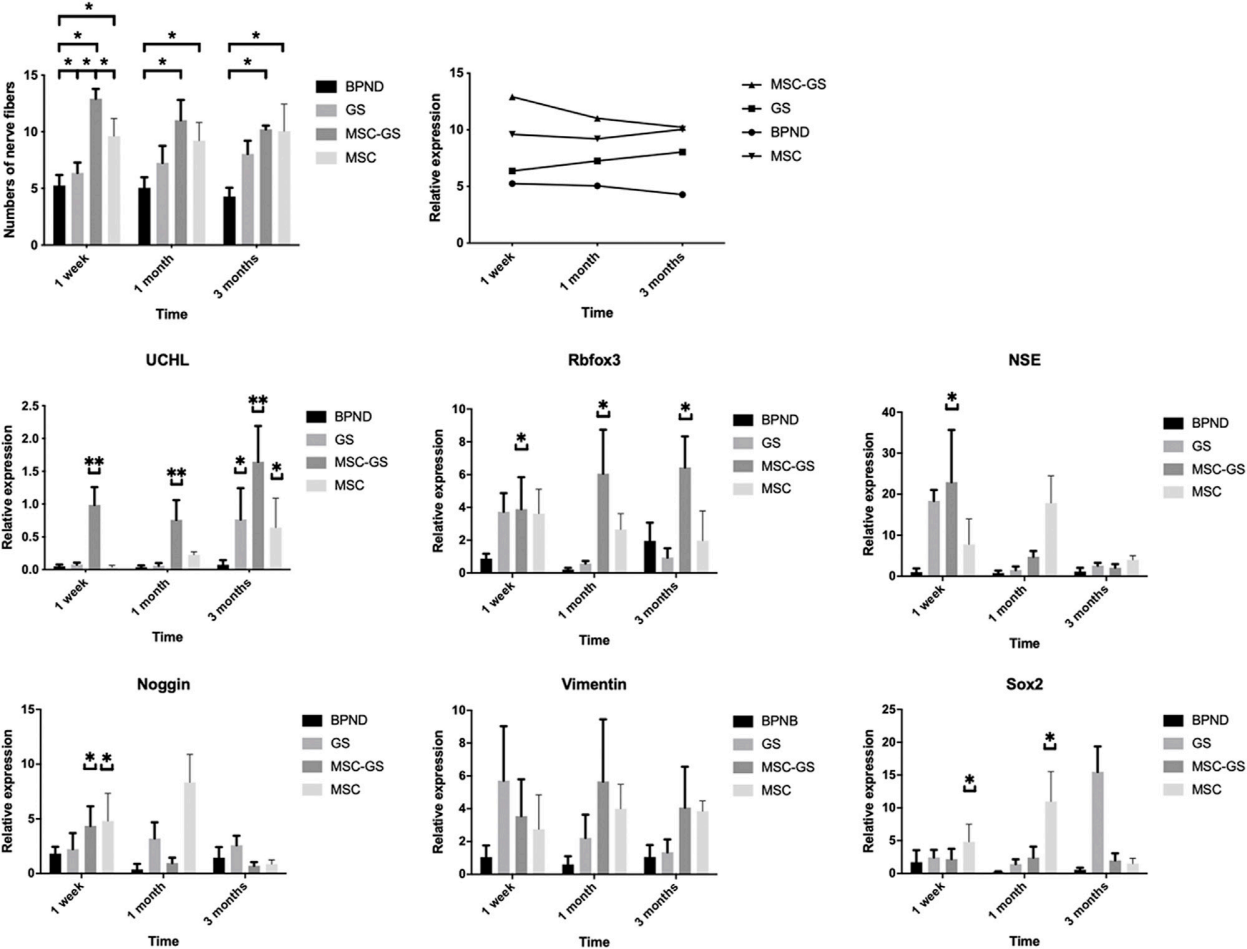
The changes of the number of nerve fibers in the anterior vaginal wall in each group at 1 week, 1 month and 3 months after operation in the bilateral pudendal nerve denervation group (BPND), blank gelatin scaffold group (GS), MSCs loaded on gelatin scaffold group (MSC-GS) and MSCs injection group (MSC). qRT-PCR was used to detect the expression of neural mRNA in each group at 1 week, 1 month and 3 months after operation. * showed that the difference was statistically significant (*p* < 0.05).

Some substances contributed to guide outgrowth of nerves. Regeneration of nerve fibers was enhanced by substances such as laminin and collagen (Vleggeert-Lankamp et al., 2004). Three methods of transplantation were evaluated: the MSC-GS group, MSC group and GS group. The numbers of nerve fibers in the anterior vaginal wall in each group at 1 week, 1 month, and 3 months after the operation are shown in Figure 6 and Supplementary Table S2. Regardless of whether the gelatin scaffold was used, MSC transplantation showed an obvious improvement of the nerve content in the anterior vaginal wall. MSCs loaded on gelatin scaffold showed more improvement, but there was no significant difference compared with MSC injection alone. The number of nerve fibers in the GS group did not change significantly 1 week after the operation but was significantly higher than that of the BPND group in the long term (1 and 3 months), suggesting that the blank gelatin scaffold could also play a certain role in nerve repair, but this effect was weak.

We also found that the gene expression of the neural-related markers UCHL, Rbfox3, NSE, and Noggin was obviously upregulated in the MSC-GS group at the early stage, among which UCHL showed the most prominent upregulation (Figure 6). Meanwhile, the expression of UCHL in the MSC group was significantly upregulated 1 month later but was still lower than that in the MSC-GS group. In the MSC-GS group, the early neuron marker proteins NSE and Noggin began to decline earlier, indicating that the whole event of nerve repair occurred earlier. In contrast, markers of neural stem cells, nestin and Sox2, increased more significantly in the early stage in the MSC group than MSC-GS group, while the expression at 3 months was still higher in the MSC-GS group, indicating that the overall repair effect in the MSC group was still better. The above data indicate that after nerve injury, the supporting role of gelatin scaffolds might be of positive significance for the maintenance of the microenvironment and strengthen the ability of MSCs to start the repair mechanism at an early stage. The reason for the high expression of neural stem cells in the early stage of MSC direct transplantation might be related to the direct exposure of MSCs to the nerve injury environment, which we will explore further in our future work.

### 3.5 Comparison of MSCs and induced neural stem cell transplantation

**FIGURE 7:**
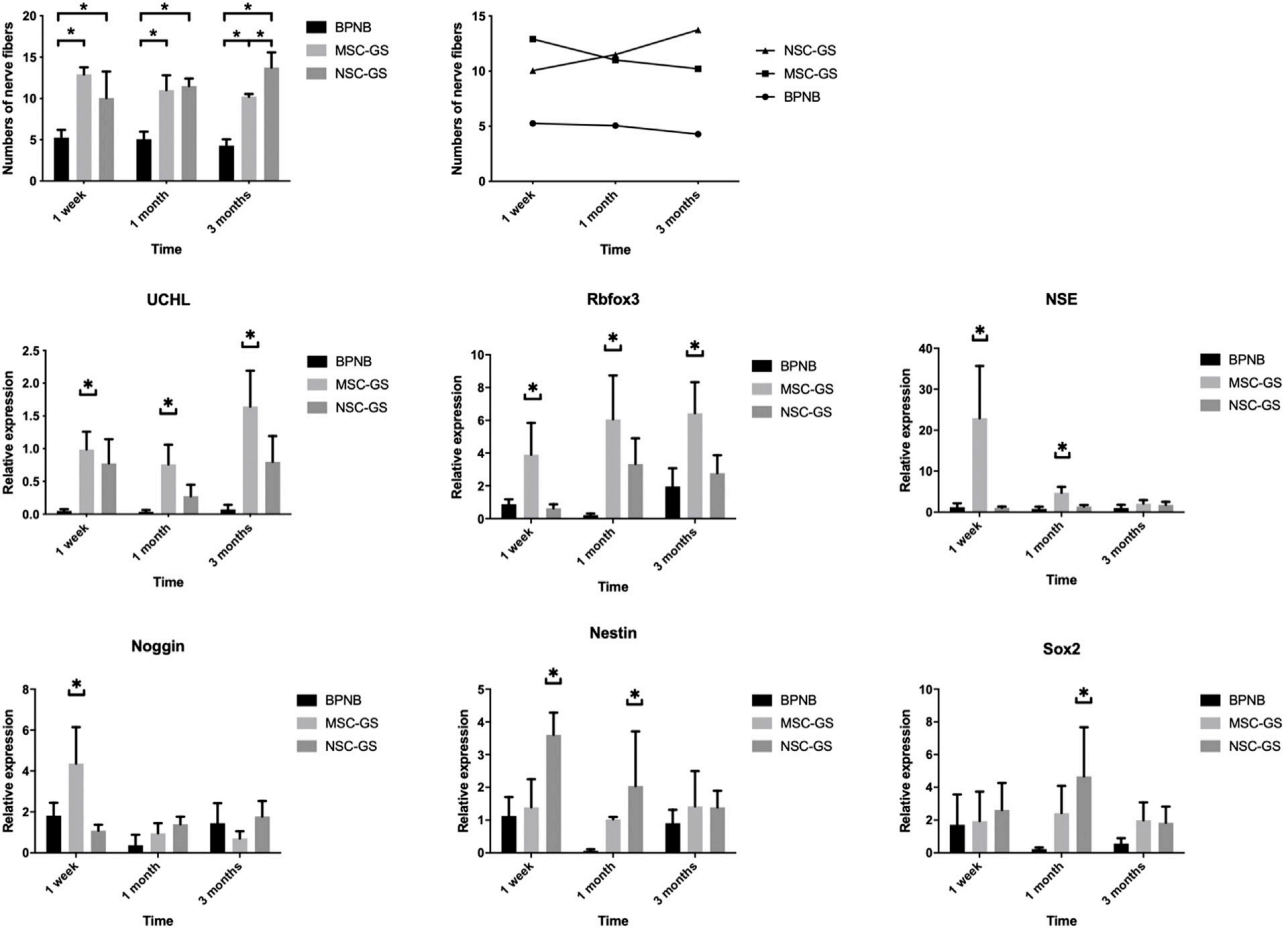
The changes of the number of nerve fibers in the anterior vaginal wall in each group at 1 week, 1 month, and 3 months after operation in the bilateral pudendal nerve denervation group (BPND), MSCs loaded on gelatin scaffold group (MSC-GS) and induced neural stem cells loaded on gelatin scaffold group (NSC-GS). qRT-PCR was used to detect the expression of neural mRNA in each group at 1 week, 1 month and 3 months after operation. * showed that the difference was statistically significant (*p* < 0.05).

We found that MSCs loaded on gelatin scaffolds were a more effective treatment method. Theoretically, it might be more effective to carry out neural induction *in vitro* first and then transplant the induced neural stem cells. Therefore, we next loaded induced neural stem cells on gelatin scaffolds for transplantation (NSC-GS group). The results (Figure 7; Supplementary Table S3) showed that the NSC-GS group tended to be superior to the MSC-GS group in improving the nerve content of the anterior vaginal wall at 1 week after the operation but had no significant advantage at 3 months. The results of gene expression analysis showed that UCHL, Rbfox3, NSE, Noggin, Nestin and Sox2 were significantly upregulated in the early stage in the NSC-GS group compared with the MSC-GS group, which also confirmed the early advantages of this scheme. The above results indicated that a combination of scaffold-based and preinduction schemes might be a more comprehensive choice for transplantation, but exploration of the specific mechanism and further verification need to be carried out in a chronic disease model more similar to that observed in clinical practice.

## 4 Discussion

The clinical application of MSCs has been widely studied (Holan et al., 2021), but rational treatment plans should be optimized for the repair of pelvic floor damage and be supported by more solid evidence. We evaluated the treatment effect of MSCs in chronic refractory tissue injury represented by PFD. With the anterior vaginal nerve injury animal model established by transecting the bilateral pudendal nerve, we compared the effects of different transplantation strategies. Our preliminary research showed that MSCs transplantation was an effective scheme for the treatment of pelvic floor nerve injury. MSCs induced in the nerve direction before transplantation had a faster onset time, and the long-term treatment effect was better after loading the MSCs on gelatin scaffolds. Our pilot study might have revealed a promising treatment scheme for pelvic floor injury.

The repair effect of MSCs has been widely confirmed, including its application in some degenerative and chronic diseases, but the existing research, generally limited to animal models, only focuses on acute injury or relatively simple models. POP animal models are mostly constructed by the spontaneous occurrence of pelvic organ prolapse after multiple deliveries, and the success of the models is only judged by naked eye observation. Wieslander (Wieslander et al., 2009) proposed the Mouse Pelvic Organ Prolapse Quantification System (MOP-Q) to judge the presence of pelvic organ prolapse and its severity. Various genetically engineered mice were used in the study of animal models of POP, including LOXL1 knockout, FBLN5 knockout and Hoxa11 knockout mice (Abramowitch et al., 2009). These POP animal models were mostly prepared by affecting the metabolism of the pelvic floor supporting structures such as collagen fibers and elastic fibers, which are the main reasons for the weakened supporting ability of pelvic floor tissue. Nerve injury is also considered one of the essential pathogeneses of POP, but there is no study on nerve injury in a POP animal model to date. In this study, an animal model of vaginal anterior wall nerve injury induced by bilateral pudendal nerve denervation provided an important basis for our research.

In degenerative diseases such as PFD, MSCs may be a potential candidate for nerve repair, in addition to their established effect in repairing connective tissue injury (Sadeghi et al., 2016). Although there is no evidence to prove that MSCs can directly differentiate into neurons, studies have shown that MSCs can quickly migrate to an injured site and differentiate into neuron-like or glial-like cells to replace the tissue-specific cell damage, which can play an alternative repair role in a short time and reduce the impairment of tissue integrity (Luo et al., 2021; Zhang et al., 2021). Moreover, MSCs can secrete many kinds of factors to promote tissue repair, cell growth and differentiation (Wang et al., 2022). In a study of retinal degeneration, MSCs mediate therapeutic effects by their potent immunoregulatory properties, inhibiting harmful inflammatory reactions, producing numerous growth and neurotrophic factors supporting the survival and growth of retinal cells, and exhibiting antiapoptotic properties. Nerve injury in PFD is a complex process, perhaps involving acute labor trauma and chronic age-related degeneration. MSC transplantation might slow the degeneration process of the damaged nerves and promote the repair of resident nerve tissue. In our study, MSCs promoted nerve repair in the anterior vaginal wall of a rat model.

In stem cell therapy, the strategy of cell transplantation and the survival of MSCs have always been key issues. A three-dimensional scaffold culture environment has a promoting effect on the clinical application of MSCs. In previous studies, it has been shown that MSCs can improve the local microenvironment by secreting cytokines, thus promoting the repair process, which may be enhanced in a three-dimensional culture environment (Raoofi et al., 2021). Scaffold pore size has been observed to influence adhesion, growth, and phenotype of cells. The optimal scaffold pore size that allows maximal entry of cells as well as cell adhesion and matrix deposition has been shown to vary with different cell types. Our preliminary study showed that GoMatrix 90 um pore size could be applied in MSCs transplantation to the anterior vaginal wall of rats. In this study, MSCs loaded on three-dimensional gelatin scaffolds showed a better and earlier onset of repair of nerve damage than direct MSC injection. Meanwhile, gene expression analysis showed that nerve regenerative capacity was upregulated, indicating a better microenvironment to maintain the growth of MSCs in three-dimensional gelatin material. However, collagenase and trypsin were not beneficial for MSCs.

The repair effect of MSCs might be achieved through direct differentiation, paracrine effect or immune regulation. Migration of MSCs to the injured site (MSCs homing) was the premise and basis for the repair effect. MSCs homing was defined as the arrest of MSCs within the vasculature of a tissue followed by transmigration across the endothelium (Karp and Leng Teo, 2009). Current MSC-based therapy for PFD usually delivers MSCs by local injection (Cheng et al., 2020). Since tissue damages in PFD were extensive, the homing property of MSCs would play a pivotal role (Sima and Chen, 2020). Ben transplanted MSCs systemically or locally to vaginal injury rat model to examine the engraftment, survival, differentiation and angiogenic effect of transplanted MSCs, and results showed both systemic and local MSCs transplantation promoted host angiogenesis (Ben Menachem-Zidon et al., 2019). In Alessio’s study, pudendal nerve was transected in female rats to induce stress urinary incontinence, and pre-differentiated human dental pulp stem cells were injected in the striated urethral sphincter, resulting in promoting vascularization and an appreciable recovery of the continence (Zordani et al., 2019). Previous studies had shown that homing was a common phenomenon in MSCs therapy. We postulated that homing phenomenon also played an important role in repairing nerve injury in pelvic floor dysfunction, however more experiments were needed to test or confirm this.

MSCs were a cluster of pluripotency stem cells derived from mesoderm, with a wide range of sources. MSCs hold the potential repair effect in tissue damage by mechanism of direct differentiation, secretion of regulatory factors and regulation of immune response. However, theoretically MSCs did not have the ability to differentiate directly into mature neural cells. This study showed MSCs had a certain repair effect on pelvic floor nerve injury, but the mechanism needed further research. Previous *in vitro* stem cell studies confirmed that MSCs could be induced to differentiate into NSCs. NSCs could directly differentiate into mature neurons and glial cells, and in this study induced NSCs had a repair effect on pelvic floor nerve injury, perhaps by direct differentiation. However, the difficulty in obtaining adequate NSCs and their low viability limited their clinical application prospects. The progress in stem cells *in vitro* research might prompt the NSCs induced from MSCs as a future strategy for stem cell transplantation in PFD.

MSCs pre-induced in the nerve direction may produce a better nerve repair effect. From the perspective of the standard developmental tree, MSCs cannot directly differentiate into real nerve cells. MSC transplantation can promote the repair of nerve injury, mainly by promoting the rescue of endogenous nerves (Luo et al., 2021; Zhang et al., 2021), but the effect is limited. We propose that MSCs induced in the nerve direction *in vitro* might have a faster effect on nerve repair *in vivo*. In our study, MSCs induced in the nerve direction had a faster repair onset time. However, the treatment effect showed no superiority in the long-term. It may be that the microenvironment in chronic diseases is not conducive to the survival of NSCs. NSCs play a nerve regeneration role but cannot survive for a long time, resulting in a declining effect. Finally, it is proposed that the best possible clinical scheme is a mixed transplantation of NSCs and MSCs, which can achieve the best therapeutic effect by multiple targets and multiple mechanisms in the repair of nerves, connective tissue and the microenvironment.

## 5 Conclusion

Above all, stem cell transplantation showed a promising repair capacity in innervation recovery of pelvic floor dysfunction. A rational comprehensive regenerative strategy requires more exploration in the future based on a better understanding of the specific mechanism and a more practical and realistic animal model. In future studies, we will further optimize the model and propose more possibilities for treating pelvic floor diseases with tissue engineering schemes.

## Data Availability

The original contributions presented in the study are included in the article/Supplementary Material, further inquiries can be directed to the corresponding authors.

## Abbreviations

MSCs, Mesenchymal stem cells; PFD, Pelvic floor dysfunction; POP, Pelvic organ prolapse; SUI, Stress urinary incontinence; PGP 9.5, protein gene product 9.5; SO, Sham operation; BPND: Bilateral pudendal nerve denervation; GAPDH, glyceraldehyde-3-phosphate dehydrogenase; NSCs, Neural stem cells; ANOVA, Analysis of variance; UCHL, Ubiquitin C-terminal hydrolase L; HPF: high- power field; MOP-Q, Mouse Pelvic Organ Prolapse Quantification System.

## References

[B1] Abramowitch S. D. Feola A. Jallah Z. Moalli P. A. (2009). Tissue mechanics, animal models, and pelvic organ prolapse: A review. Eur. J. Obstet. Gynecol. Reprod. Biol. 144 (1), S146–S158. 10.1016/j.ejogrb.2009.02.022 19285776

[B2] Ben Menachem-Zidon O. Gropp M. Ben Shushan E. Reubinoff B. Shveiky D. (2019). Systemically transplanted mesenchymal stem cells induce vascular-like structure formation in a rat model of vaginal injury. PLoS One 14, e0218081. 10.1371/journal.pone.0218081 31194823 PMC6563972

[B3] Cheng J. Zhao Z. W. Wen J. R. Wang L. Huang L. W. Yang Y. L. (2020). Status, challenges, and future prospects of stem cell therapy in pelvic floor disorders. World J. Clin. Cases 8, 1400–1413. 10.12998/wjcc.v8.i8.1400 32368533 PMC7190946

[B4] Holan V. Palacka K. Hermankova B. (2021). Mesenchymal stem cell-based therapy for retinal degenerative diseases: Experimental models and clinical trials. Cells 10, 588. 10.3390/cells10030588 33799995 PMC8001847

[B5] Hu J. M. Cheng X. Wang L. Zhu J. N. Zhou L. H. (2013). Vasoactive intestinal peptide expression in the vaginal anterior wall of patients with pelvic organ prolapse. Taiwan J. Obstet. Gynecol. 52, 233–240. 10.1016/j.tjog.2013.04.014 23915857

[B6] Hu J. M. Wang L. Cheng X. Zhou L. H. Li Z. G. (2012). Neuropeptide Y innervation in the vaginal mucosa among patients with pelvic organ prolapse. Mol. Med. Rep. 5, 444–448. 10.3892/mmr.2011.689 22143932

[B7] Karp J. M. Leng Teo G. S. (2009). Mesenchymal stem cell homing: The devil is in the details. Cell. Stem Cell 4, 206–216. 10.1016/j.stem.2009.02.001 19265660

[B8] Kuo H. (2000). The relationships of urethral and pelvic floor muscles and the urethral pressure measurements in women with stress urinary incontinence. Eur. Urol. 37, 149–155. 10.1159/000020132 10705192

[B9] Lin M. Lu Y. Chen J. (2022). Tissue-engineered repair material for pelvic floor dysfunction. Front. Bioeng. Biotechnol. 10, 968482. 10.3389/fbioe.2022.968482 36147522 PMC9485870

[B10] Luo Y. Qiu W. Wu B. Fang F. (2021). An overview of mesenchymal stem cell-based therapy mediated by noncoding RNAs in the treatment of neurodegenerative diseases. Stem Cell. Rev. Rep. 18, 457–473. 10.1007/s12015-021-10206-x 34347272

[B11] Ma Y. Zhang Y. Chen J. Li L. Liu X. Zhang L. (2021). Mesenchymal stem cell-based bioengineered constructs enhance vaginal repair in ovariectomized rhesus monkeys. Biomaterials 275, 120863. 10.1016/j.biomaterials.2021.120863 34139509

[B12] Mao M. Li Y. Zhang Y. Kang J. Zhu L. (2021). Human umbilical cord mesenchymal stem cells reconstruct the vaginal wall of ovariectomized sprague-dawdley rats: Implications for pelvic floor reconstruction. Cell. Tissue Res. 386, 571–583. 10.1007/s00441-021-03478-9 34264376

[B13] Mukherjee S. Darzi S. Paul K. Werkmeister J. A. Gargett C. E. (2019). Mesenchymal stem cell-based bioengineered constructs: Foreign body response, cross-talk with macrophages and impact of biomaterial design strategies for pelvic floor disorders. Interface Focus 9, 20180089. 10.1098/rsfs.2018.0089 31263531 PMC6597526

[B14] Raoofi A. Sadeghi Y. Piryaei A. Sajadi E. Aliaghaei A. Rashidiani-Rashidabadi A. (2021). Bone marrow mesenchymal stem cell condition medium loaded on PCL nanofibrous scaffold promoted nerve regeneration after sciatic nerve transection in male rats. Neurotox. Res. 39, 1470–1486. 10.1007/s12640-021-00391-5 34309780

[B15] Sadeghi Z. Isariyawongse J. Kavran M. Izgi K. Marini G. Molter J. (2016). Mesenchymal stem cell therapy in a rat model of birth-trauma injury: Functional improvements and biodistribution. Int. Urogynecol J. 27, 291–300. 10.1007/s00192-015-2831-5 26353846 PMC4890611

[B16] Sima Y. Chen Y. (2020). MSC-based therapy in female pelvic floor disorders. Cell. Biosci. 10, 104. 10.1186/s13578-020-00466-4 32944218 PMC7488254

[B17] Ulrich D. Edwards S. L. Su K. Tan K. S. White J. F. Ramshaw J. A. (2014). Human endometrial mesenchymal stem cells modulate the tissue response and mechanical behavior of polyamide mesh implants for pelvic organ prolapse repair. Tissue Eng. Part A 20, 785–798. 10.1089/ten.TEA.2013.0170 24083684 PMC3926142

[B18] Vergeldt T. F. Weemhoff M. Inthout J. Kluivers K. B. (2015). Risk factors for pelvic organ prolapse and its recurrence: A systematic review. Int. Urogynecol J. 26, 1559–1573. 10.1007/s00192-015-2695-8 25966804 PMC4611001

[B19] Vleggeert-Lankamp C. L. Pego A. P. Lakke E. A. Deenen M. Marani E. Thomeer R. T. (2004). Adhesion and proliferation of human Schwann cells on adhesive coatings. Biomaterials 25, 2741–2751. 10.1016/j.biomaterials.2003.09.067 14962553

[B20] Wang G. Wu H. L. Liu Y. P. Yan D. Q. Yuan Z. L. Chen L. (2022). Pre-clinical study of human umbilical cord mesenchymal stem cell transplantation for the treatment of traumatic brain injury: Safety evaluation from immunogenic and oncogenic perspectives. Neural Regen. Res. 17, 354–361. 10.4103/1673-5374.317985 34269210 PMC8463980

[B21] Wieslander C. K. Rahn D. D. Mcintire D. D. Acevedo J. F. Drewes P. G. Yanagisawa H. (2009). Quantification of pelvic organ prolapse in mice: Vaginal protease activity precedes increased MOPQ scores in fibulin 5 knockout mice. Biol. Reprod. 80, 407–414. 10.1095/biolreprod.108.072900 18987327 PMC2805390

[B22] Xu L. Sima Y. Xiao C. Chen Y. (2023). Exosomes derived from mesenchymal stromal cells: A promising treatment for pelvic floor dysfunction. Hum. Cell 36, 937–949. 10.1007/s13577-023-00887-6 36940057

[B23] Zhang M. Lin Y. H. Sun Y. J. Zhu S. Zheng J. Liu K. (2016). Pharmacological reprogramming of fibroblasts into neural stem cells by signaling-directed transcriptional activation. Cell. Stem Cell 18, 653–667. 10.1016/j.stem.2016.03.020 27133794 PMC4864020

[B24] Zhang R. C. Du W. Q. Zhang J. Y. Yu S. X. Lu F. Z. Ding H. M. (2021). Mesenchymal stem cell treatment for peripheral nerve injury: A narrative review. Neural Regen. Res. 16, 2170–2176. 10.4103/1673-5374.310941 33818489 PMC8354135

[B25] Zhu L. Lang J. Chen J. Chen J. (2004). Study on nerve fiber density in anterior vaginal epithelium for stress urinary incontinence. Int. Urogynecol J. Pelvic Floor Dysfunct. 15, 272–275. 10.1007/s00192-004-1155-7 15517673

[B26] Zordani A. Pisciotta A. Bertoni L. Bertani G. Vallarola A. Giuliani D. (2019). Regenerative potential of human dental pulp stem cells in the treatment of stress urinary incontinence: *In vitro* and *in vivo* study. Cell. Prolif. 52, e12675. 10.1111/cpr.12675 31553127 PMC6868931

[B27] Zou X. H. Zhi Y. L. Chen X. Jin H. M. Wang L. L. Jiang Y. Z. (2010). Mesenchymal stem cell seeded knitted silk sling for the treatment of stress urinary incontinence. Biomaterials 31, 4872–4879. 10.1016/j.biomaterials.2010.02.056 20303586

