# Corrigendum: Preliminary study on mesenchymal stem cells in repairing nerve injury in pelvic floor denervation

**DOI:** 10.3389/fbioe.2024.1363368

**Published:** 2024-01-12

**Authors:** Guorui Zhang, Yuxin Dai, Jinghe Lang

**Affiliations:** Department of Obstetrics and Gynecology, State Key Laboratory of Complex Severe and Rare Diseases, National Clinical Research Center for Obstetric and Gynecologic Diseases, Peking Union Medical College Hospital, Chinese Academy of Medical Sciences and Peking Union Medical College, Beijing, China

**Keywords:** mesenchymal stem cell, pelvic organ prolapse, pelvic floor dysfunction, nerve injury, stem cell transplantation

In the published article, there was an error in Figure 7 as published. The first histogram and line graph in Figure 7 were the same as **Figure 6**. The corrected Figure 7 and its caption appear below:

**FIGURE 7:**
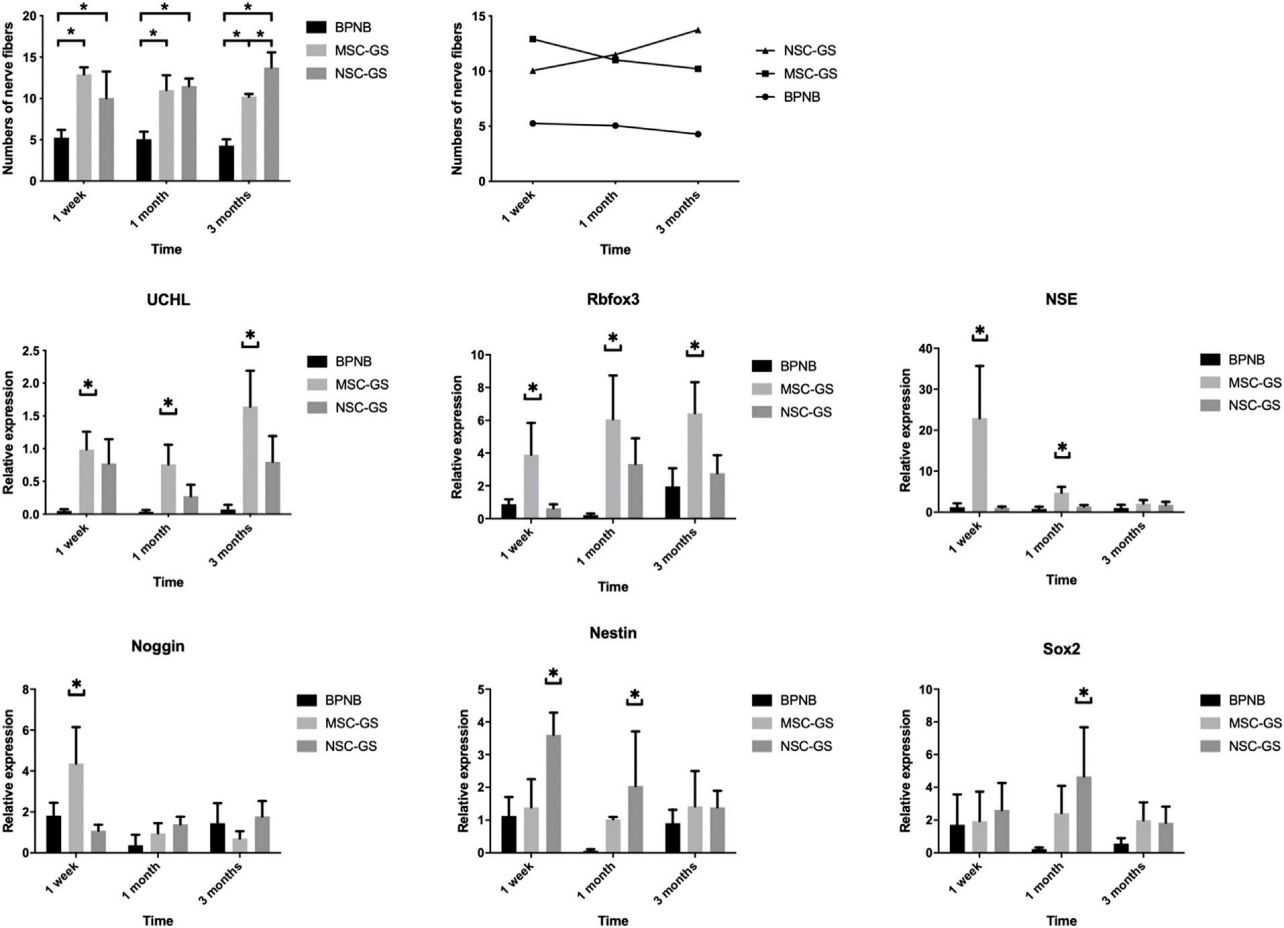
The changes of the number of nerve fibers in the anterior vaginal wall in each group at 1 week, 1 month, and 3 months after operation in the bilateral pudendal nerve denervation group (BPND), MSCs loaded on gelatin scaffold group (MSC-GS) and induced neural stem cells loaded on gelatin scaffold group (NSC-GS). qRT-PCR was used to detect the expression of neural mRNA in each group at 1 week, 1 month and 3 months after operation. * Showed that the difference was statistically significant (*p* < 0.05).

The authors apologize for this error and state that this does not change the scientific conclusions of the article in any way. The original article has been updated.

