# Supplementary material for: Preliminary study on mesenchymal stem cells in repairing nerve injury in pelvic floor denervation
Source: Front Bioeng Biotechnol. 2023 Jun 22;11:1190068. doi: 10.3389/fbioe.2023.1190068 (PMC10325727; doi:10.3389/fbioe.2023.1190068)
Supplement: Supplementary file 1 [file DataSheet1.DOCX]

Supplementary Material

Preliminary Study on Mesenchymal Stem Cells in Repairing Nerve Injury in Pelvic Floor Dernervation

**Guorui Zhang, Yuxin Dai*, Jinghe Lang**

Department of Obstetrics and Gynecology, State Key Laboratory of Complex, Severe and Rare Diseases, National Clinical Research Center for Obstetric & Gynecologic Diseases, Peking Union Medical College Hospital, Chinese Academy of Medical Sciences and Peking Union Medical College, Beijing 100730, China

*** Correspondence:**Yuxin Dai
email [helen81918@163.com](mailto:helen81918@163.com)

# Supplementary Data

**1.1 Primer sequence of qRT-PCR in animal experiment**

| Gene | Prime sequence |
| --- | --- |
| GAPDH | F: GATGGTGAAGGTCGGTGTGA |
|  | R: TGAACTTGCCGTGGGTAGAG |
| UCHL | F: ACGCAGTGGCCAATAACCAA |
|  | R: TTTGTCGTCTACCCGGCAC |
| Rbfox3 | F: CCCTCCTCCACCTCAGAATG |
|  | R: CGTGTAGAGGGTCATGCCG |
| Tubb3 | F: ATGACGACGAGGAGTCGGA |
|  | R: GGTAGGAAGGTAAGCTGGGG |
| MAP2 | F: CGAAGGAAAGGCACCACACT |
|  | R: TGGTCCTTCATCTCTGGCGA |
| NSE | F: ACGTTCATTGCAGACCTCGT |
|  | R: GAGTCGAGGTGTTCTGGGTG |
| Noggin | F: AGAAGGATCTGAACGAGACGC |
|  | R: GGGCAGAAGGTCTGTGACCA |
| Nestin | F: GACCTCCTTAGCCACAACCC |
|  | R: GTTCCCAGATTTGCCCCTCA |
| Sox2 | F: AGAACTAGACTCCGGGCGAT |
|  | R: AGAACTAGACTCCGGGCGAT |

**1.2 Names, working concentrations, molecular weights and functions of the nine small molecules used to induce mesenchymal stem cells into neural stem cells in vitro are shown in the table below.**

| Name | Concentration | Molecular weight | Function |
| --- | --- | --- | --- |
| CHIR99021 | 3uM | 465.34 | GSK-3α and β inhibitor |
| LDN193189 | 100nM | 406.48 | BMP signal transduction inhibito |
| A83-01 | 0.5uM | 421.52 | Inhibitors of ALK5, alk4 and alk7 |
| Hg-Ag 1.5 | 0.5uM | 526.04 | Hedgehog signaling pathway agonist |
| Retinoic acid | 1uM | 300.44 | Retinoic acid receptor endogenous agonist |
| SMER28 | 10uM | 264.12 | Autophagy positive regulator |
| RG108 | 10uM | 334.33 | Non-nucleoside DNA methyltransferase inhibitors |
| Parnate | 2uM | 133.19 | Inhibition of lysine specific demethylase activity |
| bFGF | 10ng/ml | * | Basic fibroblast growth factor |

**1.3 Primer sequence of qRT-PCR in neural stem induction culture**

| Gene | Primer sequence |
| --- | --- |
| GAPDH | F: GAAAGCCTGCCGGTGACTAA |
|  | R: GCATCACCCGGAGGAGAAAT |
| Sox2 | F: AAGGATAAGTACACGCTGCCC |
|  | R: GTTCATGTGCGCGTAACTGT |
| Pax6 | F: GCATTTGCATGTTGCGGAGT |
|  | R: CAGCAAAACACTTCCTCCTGC |
| Nestin | F: ACAAAGTCCCTGGCCCTCTA |
|  | R: CACTCCCCCATTCACATGCT |
| Ascl1 | F: CAGGGCTCCCGCTTCATATT |
|  | R: CAAGGAGTCGCGGAATCAGA |
| Olig2 | F: TCGCATCCAGATTTTCGGGT |
|  | R: AAAAGGTCATCGGGCTCTGG |

**2 Supplementary Figures and Tables**

**2.1 Supplementary Table 1 Numbers of nerve fibers in anterior vaginal wall in sham operation group and bilateral pudendal nerve denervation group at different times after operation**

| Group | 3 days | 1 week | 1 month | 3 months |
| --- | --- | --- | --- | --- |
| SO | 17.9±4.7 | 18.1±0.1 | 16.5±4.5 | 12.3±3.0 |
| BPND | 9.2±3.2 | 5.3±0.9 | 5.1±0.9 | 4.3±0.8 |

Abbreviations: SO, sham operation; BPND, bilateral pudendal nerve denervation.

**2.2 Supplementary Table 2 Numbers of nerve fibers in anterior vaginal wall in BPND group, GS group, MSC-GS group and MSC group at different times after operation**

| Group | 1 week | 1 month | 3 months |
| --- | --- | --- | --- |
| BPND | 5.3±0.9 | 5.1±0.9 | 4.3±0.8 |
| GS | 6.4±0.9 | 7.2±1.5 | 8.0±1.2 |
| MSC-GS | 12.9±0.9 | 11.0±1.0 | 10.2±0.3 |
| MSC | 9.6±1.6 | 9.2±1.6 | 10.0±2.4 |

Abbreviations: BPND, bilateral pudendal nerve denervation; GS, gelatin scaffold.

**2.3 Supplementary Table 3 Numbers of nerve fibers in anterior vaginal wall in BPND group, MSC-GS group and NSC-GS group at different times after operation**

| Group | 1 week | 1 month | 3 months |
| --- | --- | --- | --- |
| BPND | 5.3±0.9 | 5.1±0.9 | 4.3±0.8 |
| MSC-GS | 12.9±0.9 | 11.0±1.0 | 10.2±0.3 |
| NSC-GS | 10.1±3.2 | 11.5±0.9 | 13.8±1.8 |

Abbreviations: BPND, bilateral pudendal nerve denervation; GS, gelatin scaffold.

## 3 Supplementary Figures


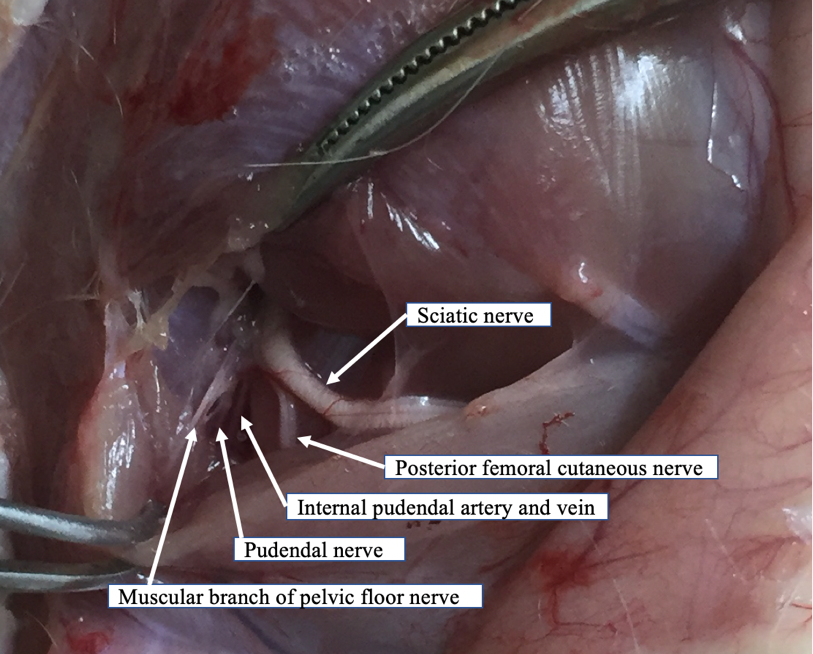


**Supplementary Figure 1.** Exposure of pudendal nerve.
